# Randomized multicentre Phase 2 clinical trial of non-viral gene therapy for pancreatic cancer: clinical and molecular findings

**DOI:** 10.20517/cdr.2026.68

**Published:** 2026-10-22

**Authors:** Louis Buscail, Marine Lebrin, Naima Hanoun, Sophie Gourgou, Noémie Dorat, Fabienne Vernejoul, Gilles Cambois, Fabian Gross, Rosine Guimbaud, Barbara Bournet, Emmanuel Mitry, Pascal Artru, Delphine Ouvrier, Nicolas Flori, Frédéric Thuillier, Marc Barthet, Michèle Tiraby, Pierre Cordelier, the Thergap-2 Investigator Group

**Affiliations:** ^1^Service de Gastroentérologie et Pancréatologie, Centre Hospitalier Universitaire de Toulouse-Rangueil (CHU) & Univ Toulouse, Toulouse 31400, France.; ^2^Module biothérapie du Centre d’Investigation Clinique (CIC 1436), INSERM & Centre Hospitalier Universitaire de Toulouse-Rangueil (CHU), Univ Toulouse, Toulouse 31400, France.; ^3^Univ Toulouse, Inserm, CNRS, CRCT, Toulouse 31100, France.; ^4^Département de Biométrie, Institut du Cancer de Montpellier, Montpellier 34090, France.; ^5^InvivoGen Company, Toulouse 31400, France.; ^6^Service d’oncologie digestive, Centre Hospitalier Universitaire de Toulouse-Rangueil (CHU) & Univ Toulouse, Toulouse 31400, France.; ^7^Oncologie Digestive, Institut Paoli-Calmettes, Marseille 13009, France.; ^8^Service d’oncologie, Hôpital Privé Jean Mermoz, Ramsay Générale de Santé, Lyon 69008, France.; ^9^Service de Gastroentérologie, Centre Hospitalier Universitaire de Nice-L’archet (CHU) & Univ Nice, Nice 06200, France.; ^10^Gastroentérologie et Nutrition clinique, Institut du Cancer de Montpellier, Montpellier 34090, France.; ^11^Service d’Oncologie Digestive, Centre Hospitalier Universitaire de Limoges-Dupuytrens (CHU) & Univ Limoges, Limoges 87000, France.; ^12^Service de Gastroentérologie, Centre Hospitalier Universitaire Nord (CHU), APHM & Univ Marseille, Marseille 13005, France.

**Keywords:** Pancreatic cancer, chemotherapy, resistance, gene therapy, clinical trial, clusterin

## Abstract

**Aim:** Pancreatic ductal adenocarcinoma (PDAC) has a dismal prognosis, and innovative therapies are needed. We developed a phase I pilot, non-viral gene therapy (CYL-02) encoding *SST2* and *DCK::UMK* genes complexed with a polyethyleneimine non-viral vector to sensitize tumours to gemcitabine chemotherapy, which showed excellent feasibility and safety.

**Methods:** An open-label, multicentre, randomized Phase 2 trial (NCT02806687) compared CYL-02 [two intratumoral endoscopic ultrasound (EUS)-guided injections one month apart] + gemcitabine injected weekly *vs*. gemcitabine alone in 70 patients (*n* = 35 per arm) with locally advanced PDAC. The primary endpoint was progression-free survival (PFS). Secondary endpoints were overall survival (OS), tumour response, safety, and ancillary studies.

**Results:** PFS did not differ between arms. Unexpectedly, OS was worse in the gene therapy + chemotherapy arm. Exploratory analyses revealed no differences in clinical status, baseline characteristics, or the number of patients receiving second-line therapy, with the exception of a more frequent use of FOLFOX post-progression in the control arm, which could explain this difference. Proteomic profiling identified clusterin (CLU) as a candidate treatment-induced resistance factor in patients: CYL-02 + gemcitabine significantly increased circulating CLU levels, while gemcitabine alone did not. *In vitro* and *in vivo* models confirmed CLU induction by DCK combined with gemcitabine and demonstrated that CLU silencing restored chemosensitivity, reducing experimental tumour volume in mice.

**Conclusion:** CLU induction in patients receiving CYL-02 and gemcitabine may explain adaptive resistance and lack of efficacy of gene therapy. These results provide a rationale for combining CYL-02 and gemcitabine with CLU targeting to overcome resistance in pancreatic cancer.

## INTRODUCTION

Pancreatic ductal adenocarcinoma (PDAC) is the fifth leading cause of cancer-related deaths in Western countries and is projected to become the second leading cause of cancer-related death within 5 years^[1,2]^. With curative surgical resection feasible in only 10%-18% of patients, the majority present with locally advanced or metastatic disease, for which systemic chemotherapy remains the only option^[1]^. Despite significant advances in immunotherapy for other malignancies, PDAC remains largely refractory to immune checkpoint inhibitors (e.g., anti-PD-1/PD-L1) due to its immunologically “cold” tumour microenvironment, characterized by sparse T-cell infiltration, abundant immunosuppressive stromal components, and intrinsic resistance mechanisms^[3]^. The emergence of RAS-on inhibitors has demonstrated encouraging clinical activity in PDAC, offering a novel approach to overcome the long-standing challenge of targeting this historically undruggable oncogene^[4]^. In a palliative setting, current standards of care, gemcitabine, FOLFIRINOX, or gemcitabine plus nab-paclitaxel, provide only modest survival benefits [median overall survival (OS) of 6-11 months] and are ultimately limited by the inevitable development of resistance^[5-8]^.

To overcome these challenges, gene therapy strategies have emerged as promising tools to defeat treatment resistance, employing a variety of molecular approaches, including plasmid DNA, siRNA, and CRISPR-Cas9-based systems. Interestingly, Huang *et al.* recently developed an innovative approach targeting the PDAC stroma using the LQT peptide, which selectively binds to fibronectin 1 in pancreatic stellate cells. This peptide was used to generate a lipid-dendrimer nanoparticle for the targeted delivery of interleukin-2 plasmid DNA, resulting in amplified CD8+ T-cell infiltration and activation. This strategy led to significant suppression of PDAC progression when combined with checkpoint inhibitors, such as anti-PD-1 antibodies, or co-stimulatory molecules, like the OX40 ligand^[9]^. Another compelling example involves the use of virus-inspired nanoparticles to deliver siRNA targeting signal transducer and activator of transcription 3 (STAT3). These nanoparticles exploit filopodia-mediated internalization and, when combined with gemcitabine, strongly suppress experimental tumour growth^[10]^. A similar STAT3-targeting strategy, using lipid conjugates, combined with *Cd274*, which encodes PD-L1, sensitized PDAC models to standard-of-care immune checkpoint inhibitors^[11]^. Additionally, CRISPR-Cas9-mediated gene editing of Sirpα and Siglec10 in macrophages, using a poly(β-amino ester) cationic polymer, has been shown to stimulate antitumour immune responses and reduce tumour volume in murine models, including patient-derived PDAC xenografts in humanized mice^[12]^. Regarding viral vectors, a recent phase I/II clinical trial evaluated LOAd703, an oncolytic adenovirus designed as a tumour microenvironment gene engineering vector. LOAd703 encodes genes targeting the CD40 and 4-1BB pathways, effectively inflaming “cold” PDAC tumours and inducing a significant upregulation of Th1 immunity biomarkers^[13]^.

In this context, we developed a non-viral gene therapy platform using polyethyleneimine (PEI) as a vector to deliver a plasmid encoding two therapeutic transgenes: *SSTR2* (somatostatin receptor subtype 2, SST2) and *DCK::UMK* (a fusion of deoxycytidine kinase and uridyl monophosphate kinase)^[14]^. This dual-gene strategy was rationally designed to inhibit cancer cell biology and to enhance the efficacy of gemcitabine through two complementary mechanisms. First, SST2 exerts anti-proliferative and anti-secretory effects via somatostatin signaling pathways, thereby inhibiting the cancer cell cycle, promoting apoptosis, and reducing angiogenesis and metastatic spread *in vivo*^[15]^. Second, the DCK::UMK fusion enhances the intracellular activation of gemcitabine, which requires phosphorylation to exert its cytotoxic effects, thereby inducing the death of PDAC cells both *in vitro* and *in vivo*^[16]^. Specifically, the combination of UMK, now named UMPCK for Uridine Monophosphate Cytidine Kinase, with DCK expression was motivated to counteract the lability of gemcitabine’s intermediate metabolites in the tumour microenvironment. This lability is due to the activity of cytidine deaminase and deoxycytidylate deaminase, two enzymes whose activities bypasses the phosphorylation pathway of gemcitabine, thereby preventing its conversion into the cytotoxic metabolite dFdCTP^[17]^. Extensive preclinical studies in mouse and hamster PDAC models demonstrated that intratumoural delivery of these genes produced significant inhibition of tumour growth^[14]^, establishing a strong rationale for clinical translation.

These preclinical results led to a Phase I pilot gene therapy clinical trial (Thergap-1, NCT01274455)^[18]^. In this study, patients with unresectable PDAC received two endoscopic ultrasound (EUS)-guided intratumoral injections of CYL-02, a GMP-grade plasmid encoding *SST2* and *DCK::UMK* complexed with PEI non-viral vector, at escalating doses (125-1,000 µg), followed by gemcitabine therapy. The procedure was feasible and well tolerated, with no dose-limiting toxicities or serious adverse events (SAEs) directly attributable to CYL-02, regardless of tumour location or prior treatment^[18]^. In the subgroup of patients with locally advanced pancreatic cancer and naïve to treatment, encouraging efficacy signals emerged: the median progression-free survival (PFS) and OS were 5.75 and 12.2 months, respectively, with the notable observation that 11 of 12 patients did not develop metastatic disease during follow-up^[18]^. These promising results provided the foundation for a randomized, multicentre Phase 2 trial (Thergap-2; ClinicalTrials.gov identifier NCT02806687) to rigorously assess whether the addition of CYL-02 to standard gemcitabine chemotherapy could improve outcomes in previously untreated patients with locally advanced, non-metastatic PDAC.

## METHODS

### Patients

Inclusions started in January 2017 and ended in January 2021, with a 6-month interruption in 2020 due to the COVID-19 pandemic. Inclusion criteria required histologically or cytologically confirmed unresectable PDAC with a measurable lesion per RECIST v1.1, age ≥ 18 years, absence of liver metastases confirmed by systematic MRI, no prior antitumor therapy, no contraindications to EUS examination or chemotherapy, obstructive jaundice adequately managed, and Eastern Cooperative Oncology Group (ECOG) performance status ≤ 2. Exclusion criteria included metastatic disease (except isolated pulmonary nodules < 10 mm), severe comorbidities, or pregnancy/breastfeeding.

### Trial design and oversight

The Thergap-2 trial was a prospective, open-label, multicentre, randomized Phase 2 study (ClinicalTrials.gov identifier: NCT02806687; EUDRACT: 2016-000572-20) conducted across seven French centres (Limoges, Lyon, Marseille, Montpellier, Nice, and Toulouse [coordinating centre]). The trial was approved by the Ethical Committee (Comité de Protection des Personnes Sud Ouest et Outre Mer II, CPP File No. 2-16-11) on June 2, 2016, and by the French National Agency for Medicines and Health Products Safety (ANSM, #1605-42A-41) on December 20, 2016. All patients provided written informed consent prior to enrolment, in accordance with the Declaration of Helsinki and Good Clinical Practice guidelines. Toulouse University Hospital (CHU Toulouse) sponsored the trial, and the trial design is shown in Figure 1. Patients were randomized 1:1 to Arm A (CYL-02 + gemcitabine) or Arm B (gemcitabine alone) using the Clinsight Software (CSRandom module, Biometric Department of the Montpellier Cancer Institute). Randomization was stratified by centre, ECOG performance status (0-1 *vs.* 2), and age (< 65 *vs.* ≥ 65 years). All clinical, biological, and radiological data were centralized via an electronic case report form (Ennov Clinical Software) and independently validated by the Biometric Department of the Montpellier Cancer Institute for statistical analysis.

**Figure 1:**
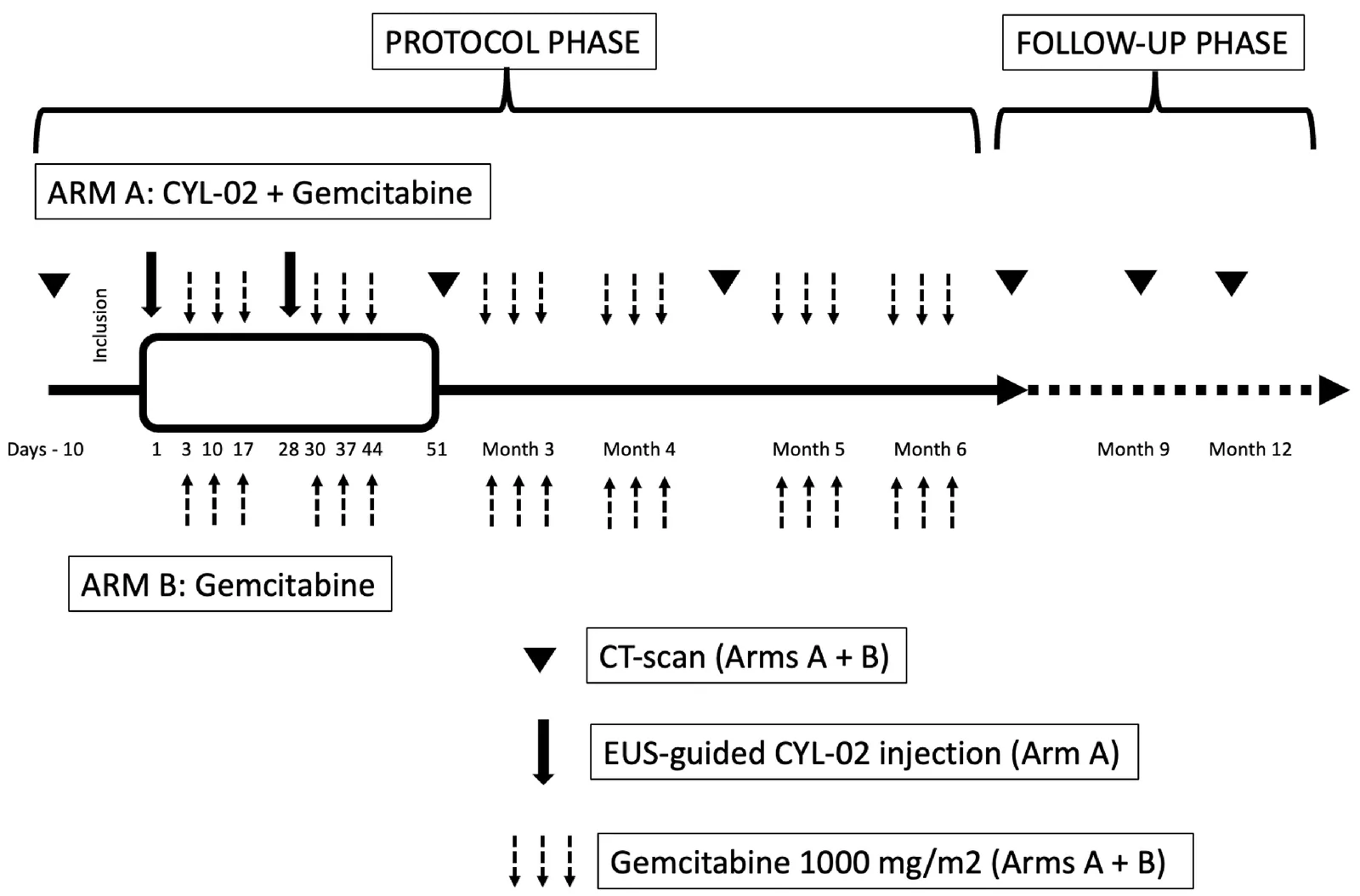
Study design of the Thergap-2 clinical trial for locally advanced pancreatic ductal carcinoma. The schematic illustrates the timeline and interventions of the Thergap-2 protocol, divided into two phases: the Protocol Phase and the Follow-Up Phase. Protocol phase: patients with locally advanced PDAC were enrolled during the pre-treatment period (Days -10 to 1). In Arm A (CYL-02 + gemcitabine), patients received CYL-02 via EUS-guided injection on Day 1 and Day 28, followed by gemcitabine (1,000 mg/m^2^) on specified days (Days 3, 10, 17, 28, 37, and 44). In Arm B (gemcitabine), patients received gemcitabine (1,000 mg/m^2^) alone on the same schedule as Arm A. CT scans were performed for both Arms at the indicated time points to monitor disease progression. The follow-up phase began after the 6-month Protocol phase or at the time of disease progression and continued until the patient’s death, with regular follow-up assessments. Symbols: solid arrows: CYL-02 injections (Arm A only). Dashed arrows: gemcitabine administrations (Arms A + B). Triangle: CT scans (Arms A + B). PDAC: Pancreatic ductal adenocarcinoma; EUS: endoscopic ultrasound; CT: computed tomography.

### Interventions

CYL-02 was provided by InvivoGen (Toulouse, France) after an external validation using *in vitro* and *in vivo* tests for purity, stability, and biological activity as previously described^[14]^. Patients from Arm A received two intratumoral injections of CYL-02 (D1 and D28) via EUS guidance. The lyophilized product (1,000 µg) was reconstituted in 2.5 mL of isotonic 5% glucose solution 5 min prior to injection. The injection protocol was standardized across centres, with real-time visualization of a white cloud confirming accurate intratumoral targeting^[18]^. Patients from both Arms Received gemcitabine (1,000 mg/m^2^ weekly), 3 weeks on/1 week off, for 6 months (follow-up phase) or until progression. For patients in Arm A, gemcitabine (1,000 mg/m^2^) was administered 48 h after CYL-02 injection. For the post-progression phase, subsequent treatments were determined by local multidisciplinary teams per standard practice.

### Endpoints and assessments

The primary endpoint was PFS, assessed by clinical signs of progression and/or serial CT scans. The secondary endpoints were OS, feasibility in terms of technical success rate of EUS-guided CYL-02 intratumoral injections, antitumor response assessed by EUS at D28 (Arm A only) and serial CT scans (both Arms) per RECIST v1.1, biomarker dynamics with CA 19-9 serum levels monitoring at baseline, M2, M4, and M6 post intervention, quality of life using EORTC QLQ-C30 questionnaire at baseline and M2, and safety, with adverse events (AEs) graded per CTCAE v4.03, and serious AEs reviewed by an independent safety committee at midterm.

### Ancillary studies

#### Sample collection and processing

Blood samples were collected from Thergap-1 and Thergap-2 patients at CHU Toulouse at baseline and post-treatment time points (D1, D28, M2, M4) as previously described^[18]^. Plasma (50 µL) was processed using ProteoMiner kits (Bio-Rad) for protein equalization, followed by elution in Laemmli buffer, reduction, and alkylation. Proteins were separated by SDS-PAGE and in-gel digested with trypsin.

#### Proteomic analysis

Peptides from two biological samples collected from the same patient were analyzed via nano-liquid chromatography (Agilent Chip-Cube) coupled to an electrospray ionization mass spectrometer (Bruker Amazon ETD) as previously described^[18]^. Briefly, raw files were processed using Progenesis LC-MS (v4.1) and searched against the SwissProt database via MASCOT. Statistical filtering: Proteins with ≥ 2 unique peptides and false discovery rate (FDR) < 1% (Benjamini-Hochberg correction) were retained. Differentially expressed proteins were identified using analysis of variance (ANOVA) with FDR correction (q < 0.05). Prioritization criteria: proteins with ≥ 2-fold increase and *P* < 0.05 (after correction) were selected for validation.

#### Clusterin levels quantification by ELISA

Clusterin (CLU) levels in plasma (25 µL) were quantified using Quantikine ELISA (R&D Systems).

#### Lentiviral vector cloning and production

The DCK open-reading frame (Gene ID: 1633) was cloned into pCDH-CMV-MCS-EF1-CopGFP cDNA (System Biosciences, CD511-B1) and validated by Sanger sequencing. Control and CLU-targeting shRNAs (GeneCopoeia) were used to generate lentiviral vectors using standard procedures.

#### Cell culture and cell confluence analysis

The human pancreatic cancer cell lines MIA PaCa-2 (CRL-1420, ATCC), BxPC-3 (CRL-1687, ATCC), and AsPC-1 (CRL-1682, ATCC) were obtained from the American Type Culture Collection (ATCC). All cell lines were authenticated by short tandem repeat (STR) profiling and tested weekly for mycoplasma contamination using the MycoAlert Mycoplasma Detection Kit (LT07-318, Invivogen). Experiments were conducted using cells at passages below 10 to ensure phenotypic stability. MIA PaCa-2 cells were cultured in DMEM supplemented with 10% fetal bovine serum (FBS). BxPC-3 cells and ASPC-1 cells were maintained in RPMI-1640 medium supplemented with 10% FBS. All cell lines were incubated at 37 °C in a humidified atmosphere containing 5% CO_2_. MIA PaCa-2 cells were transduced to generate control, DCK-overexpressing, or CLU-silenced cells following standard procedures. For treatments, cells were exposed to 100 nM gemcitabine, while phosphate-buffered saline (PBS) was used as a mock treatment. Cell counts were determined using the Coulter Z1 Particle Counter (Beckman Coulter Life Sciences), and cell confluence was monitored in real-time using the IncuCyte Zoom system (Sartorius).

#### Mouse model

MIA PaCa-2 cells (2 × 10^6^) were orthotopically engrafted into the pancreas of 6-week-old female NSG [NOD scid, *Prkdc* mutation, and a complete null allele of the IL2 receptor common gamma chain (*IL2rg^null^*)] mice (Charles River), which were randomized at the time of cancer cell implantation. (Protocol #: APAFIS#57814-2025092411253403v9). Mice received 135 mg/kg gemcitabine or placebo (PBS) every 2-3 days for 9 days. Tumour progression was tracked via bioluminescence imaging (IVIS Spectrum, Perkin Elmer) after D-luciferin injection. The experimenters were blinded to both the type of implanted cells and the treatment (placebo or gemcitabine).

#### Immunohistochemistry

Tumours were formalin-fixed, paraffin-embedded, and sectioned (4 µm). Antigen retrieval was performed using Sodium Citrate Buffer (10 mM Sodium Citrate, 0.05% Tween 20, pH 6.0) and Citric Acid Buffer (10 mM Citric Acid, 0.05% Tween 20, pH 6.0). Nonspecific signal was blocked with a blocking reagent (DakoCytomation, Santa Clara, CA, USA). Slides were incubated overnight at 4 °C with Anti-Ki67 [SP6] (ab16667, Abcam) diluted in Antibody diluent solution (DakoCytomation, Santa Clara, CA, USA). Endogenous peroxidase activity was quenched using H_2_O_2_. EnVision+ System- HRP labelled polymer anti-rabbit was added (DakoCytomation, Santa Clara, CA, USA), followed by horseradish peroxidase streptavidin (dilution 1:500, SA-5004, Vector, Burlingame, CA, USA). Slides were washed in PBS, incubated in AEC+ reagent, and counterstained with Mayer’s hematoxylin (DakoCytomation). After PBS wash, slides were mounted with Vectashield (Vector Labs, Newark, CA, USA). Images were acquired with an AXIO optical microscope (Zeiss, Göttingen, Germany) equipped with a colour AXIOCAM camera and quantified using ImageJ.

#### Western Blot analysis

Culture medium was purified using Vivaspin 20 columns (Cytiva, Marlborough, MA, USA). Cellular proteins were extracted using RIPA buffer (50 mM Tris-HCl pH 8, 150 mM NaCl, 0.5% NP40, 0.5% sodium deoxycholate, 0.1% SDS). Proteins were separated by SDS-PAGE and blotted for CLU (D4B6P, Cell Signaling Technology, Danvers, MA, USA), DCK (ab38012, Abcam, Cambridge, UK), and β-actin (Cell Signaling Technology, 8H10D10). HRP conjugate secondary antibodies were purchased from Promega, and signals were detected using ECL (Bio-Rad, Hercules, CA, USA), acquired using the ChemiDoc system (Bio-rad) and quantified with Image Lab (Bio-Rad). CLU levels in plasma (25 µL) were measured via Quantikine ELISA (R&D Systems Techne, Minneapolis, MN, USA).

#### Statistical analysis

Clinical data Statistical analysis was performed using SAS software (Version 9.4, SAS Institute, Cary, North Carolina, USA). Survival analyses: Kaplan-Meier curves + log-rank tests; HRs and 95%CIs via Cox models, adjusted for age, ECOG performance status, and tumour location. Subgroup analyses: Forest plots with interaction tests. Categorical data: Fisher’s exact test or chi-square test. Continuous data: Student’s *t*-test or Mann-Whitney test. Correlation analyses: Spearman’s rank correlation.

Ancillary data Statistical analysis was performed using GraphPad Prism version 10.6.1 (799), September 8, 2025, for Mac (GraphPad Software, Boston, Massachusetts, USA, www.graphpad.com). Proteomics: ANOVA with Benjamini-Hochberg FDR correction (q < 0.05). ELISA: Paired/unpaired *t*-tests or Mann-Whitney tests. *In vitro*: Two-way ANOVA + Tukey’s post-hoc tests. *In vivo*: Two-way ANOVA. All tests were two-sided, with *P* < 0.05 considered significant unless stated otherwise. Values are presented as ^*^P < 0.05, ^**^P < 0.01, ^***^P < 0.001, ^****^P < 0.0001. Error bars are S.D. unless otherwise stated.

## RESULTS

### Patient characteristics and protocol procedures

Figure 1 illustrates the Thergap-2 trial design. In brief, patients were randomized 1:1 in an open-label design to Arm A (CYL-02 + gemcitabine) or Arm B (gemcitabine alone), and randomization was stratified by centre, ECOG performance status (0-1 *vs*. 2), and age group (< 65 *vs.* ≥ 65 years).

The baseline patient characteristics are detailed in Table 1, displaying no difference between Arm A (CYL-02 + gemcitabine) and Arm B (gemcitabine alone). In addition, there were no differences in various comorbidities, including nutritional disorders or venous thrombosis, at baseline. The CONSORT Flow diagram is shown in Figure 2. The independent safety committee reviewed the first 50 patients in July 2020 and approved continuation.

**Figure 2:**
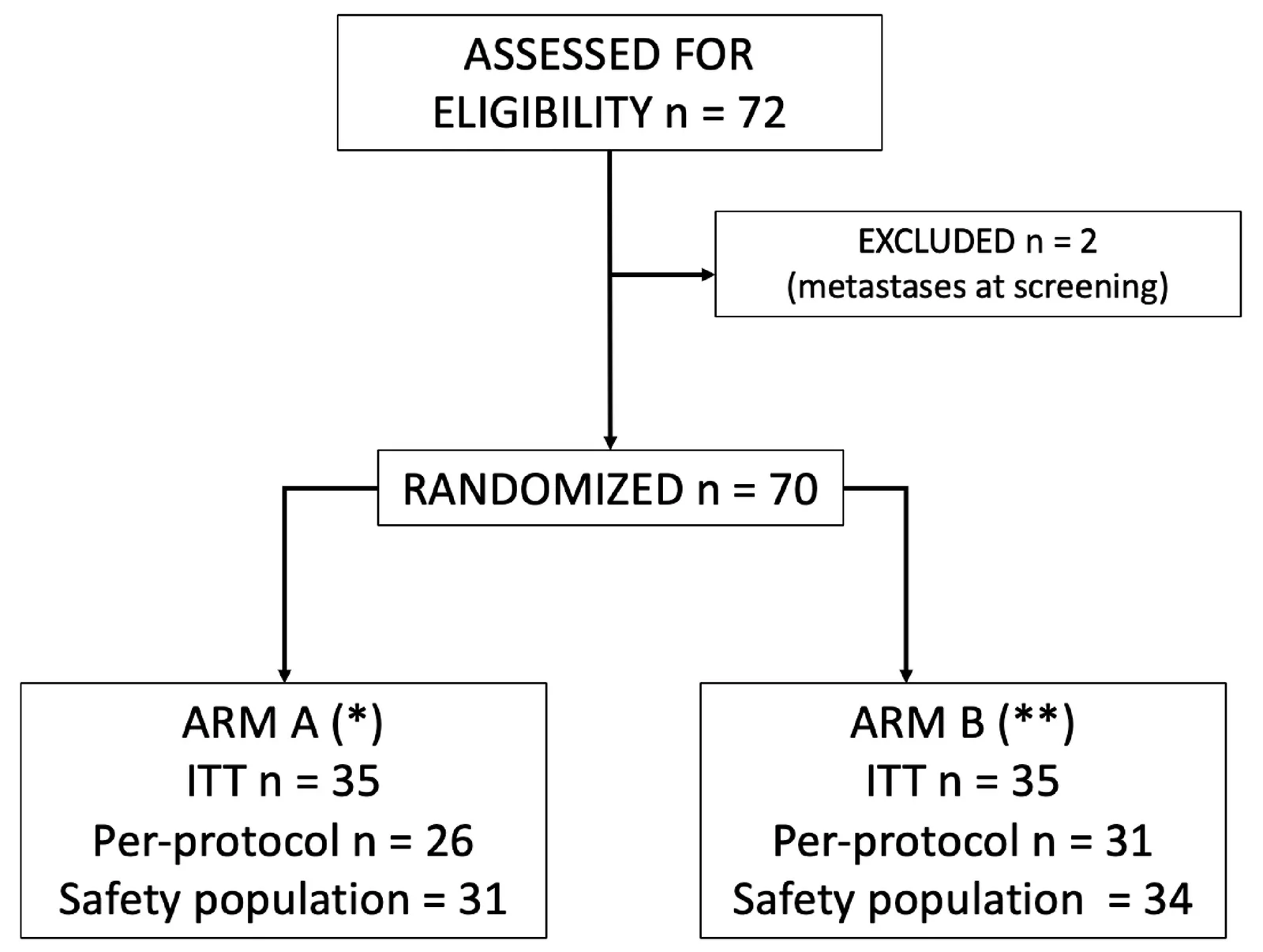
CONSORT flow-chart of the Thergap-2 clinical protocol of gene therapy for locally advanced PDAC. This flow-chart illustrates the progression of patients through the Thergap-2 clinical trial. Seventy-two patients were initially assessed for eligibility. Two patients were excluded due to the presence of metastases at screening. Seventy patients were randomized into two arms: Arm A (*): thirty-five patients were included in the ITT population. Twenty-six patients adhered to the protocol without violations of inclusion and exclusion criteria, receiving at least 3 weeks of gemcitabine and two CYL-02 intratumoural injections. Thirty-one patients were evaluable for tolerance and received at least one administration of the treatments. (*): Four patients did not receive allocated treatment due to the following reasons: borderline tumor (*n* = 1), pelvic fracture with inability to perform endoscopic ultrasound, persistent thrombocytopenia with grade 2 portal hypertension, and inability to inject CYL-02 due to pyloric suture after surgical digestive bypass. Arm B (**): thirty-five patients were included in the ITT population. Thirty-one patients adhered to the protocol without violations of inclusion and exclusion criteria, receiving at least 3 weeks of gemcitabine. Thirty-four patients were evaluable for tolerance and received at least one administration of the treatment. (**): One patient did not receive the allocated treatment because the patient withdrew consent. Definitions: ITT includes all randomized patients, treated or not, eligible or not. Per-Protocol includes all patients without violation of inclusion and exclusion criteria and receiving at least 3 weeks of gemcitabine (Arms A and B) as well as two CYL-02 intratumoural injections (Arm A). Safety includes all patients evaluable for tolerance and who received at least one administration of either treatment. PDAC: Pancreatic ductal adenocarcinoma; ITT: intent-to-treat.

**Table 1:** Baseline characteristics of patients included in the Thergap-2 trial

**Characteristics**	**Arm A** **CYL-02 + gemcitabine** ***N* = 35**	**Arm B** **Gemcitabine** ***N* = 35**	***P***
Age (median-range) < 65 years 65 years	72 (53-88) 10 25	69 (46-88) 11 24	1.0
Gender F M	16 (45.7%) 19 (54.2%)	19 (54.2%) 16 (45.7%)	0.63
ECOG score 0-1 2	29 (82.8%) 6 (17.1%)	30 (85.7%) 5 (14.2%)	1.0
Tumour location Head Body Tail	25 (71.4%) 9 (25.7%) 1 (2.8%)	21 (60.0%) 12 (34.2%) 2 (5.7%)	0.80
Tumour size (mm)* Median (range)	30 (16-50)	27 (13-58)	0.27
Previous interventions** Surgical bypass Biliary stenting	5 (14.2%) 20 (57.1%)	7 (20.0%) 18 (51.4%)	0.74
Route of EUS-guided CYL-02 injection*** Transduodenal Transgastric	19 (61.3%) 12 (38.7%)	-	-

*Maximal diameter at baseline CT scan. **10 Patients in each Arm A and B did not have previous interventions. ***Four patients did not receive allocated treatment for Arm A: borderline tumour (*n* = 1), pelvic fracture with inability to perform endoscopic ultrasound, persistent thrombocytopenia with grade 2 portal hypertension, inability to inject CYL-02 due to pyloric suture after surgical digestive bypass (i.e., a total of 31 patients were injected). Mann-Whitney and Fisher’s exact tests were used to determine the statistical significance of differences between the two patient groups. ECOG: Eastern Cooperative Oncology Group; EUS: endoscopic ultrasound; CT: computed tomography.

### Primary endpoints

There was no significant difference in PFS between the two Arms (hazard ratio = 1.16; *P* = 0.6); therefore, the primary endpoint was not met. Figure 3 shows the Kaplan-Meier curves for the intent-to-treat (ITT) population, with consistent results observed in the treated and per-protocol populations [Table 2]. On the contrary, the OS analysis revealed a statistically significant difference favouring Arm B (gemcitabine alone) over Arm A (CYL-02 + gemcitabine, Figure 4 and Table 2). Specifically, the median OS for Arm A was 12.8 months (treated population) and 13.2 months (ITT population), compared to 17.5 months (treated) and 17.8 months (ITT) for Arm B, with hazard ratios of 1.83 (95%CI: 1.06-3.16) for the treated population and 1.82 (95%CI: 1.06-3.14) for the ITT population, respectively.

**Figure 3:**
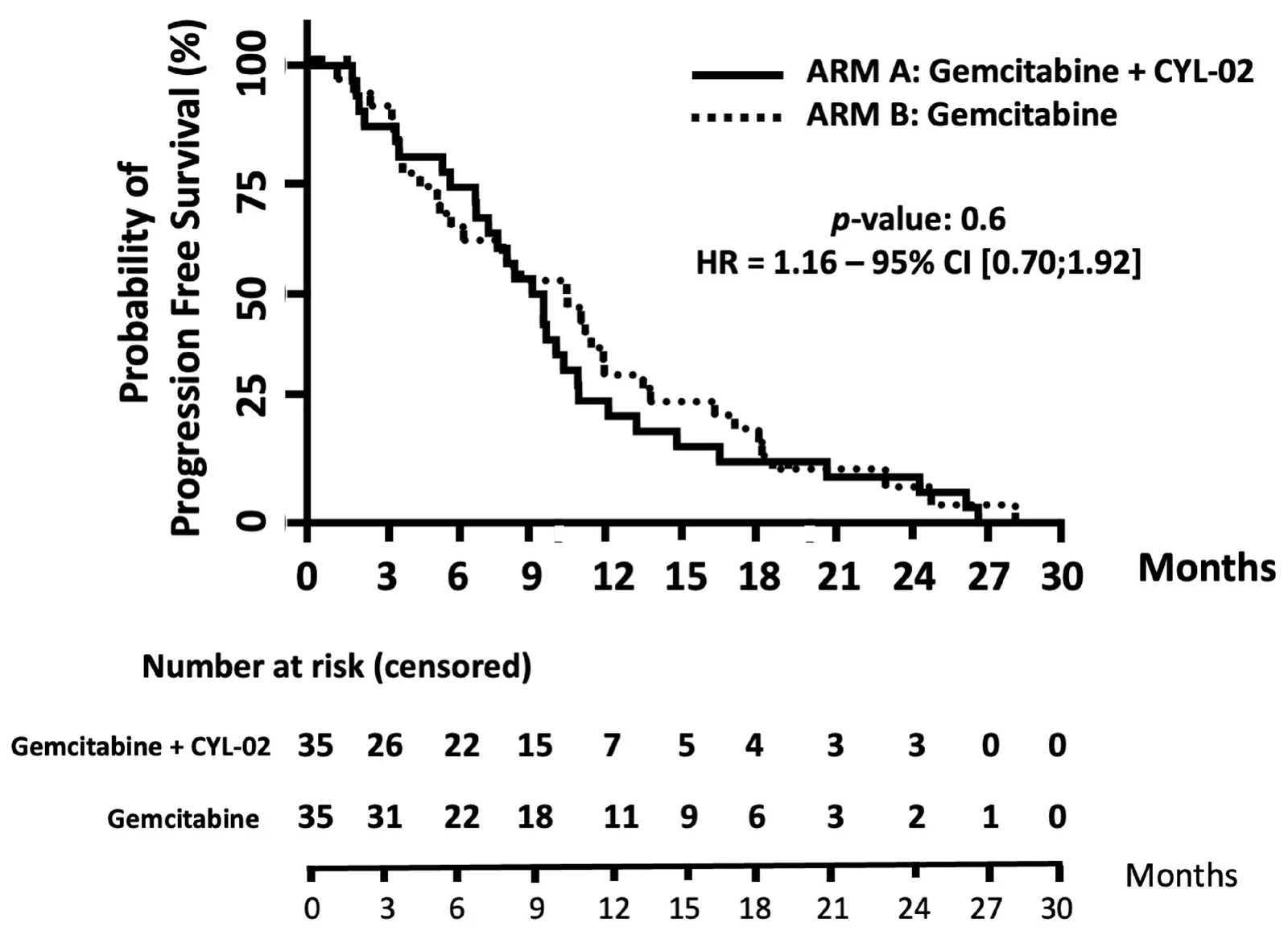
PFS analysis using Kaplan-Meier curves for the ITT population of patients with locally advanced PDAC. This figure presents PFS probabilities over 30 months for patients treated with CYL-02 + gemcitabine (Arm A, continuous line) and gemcitabine alone (Arm B, dotted line). PFS: Progression-free survival; ITT: intent-to-treat; PDAC: pancreatic ductal adenocarcinoma; HR: hazard ratio; CI: confidence interval.

**Figure 4:**
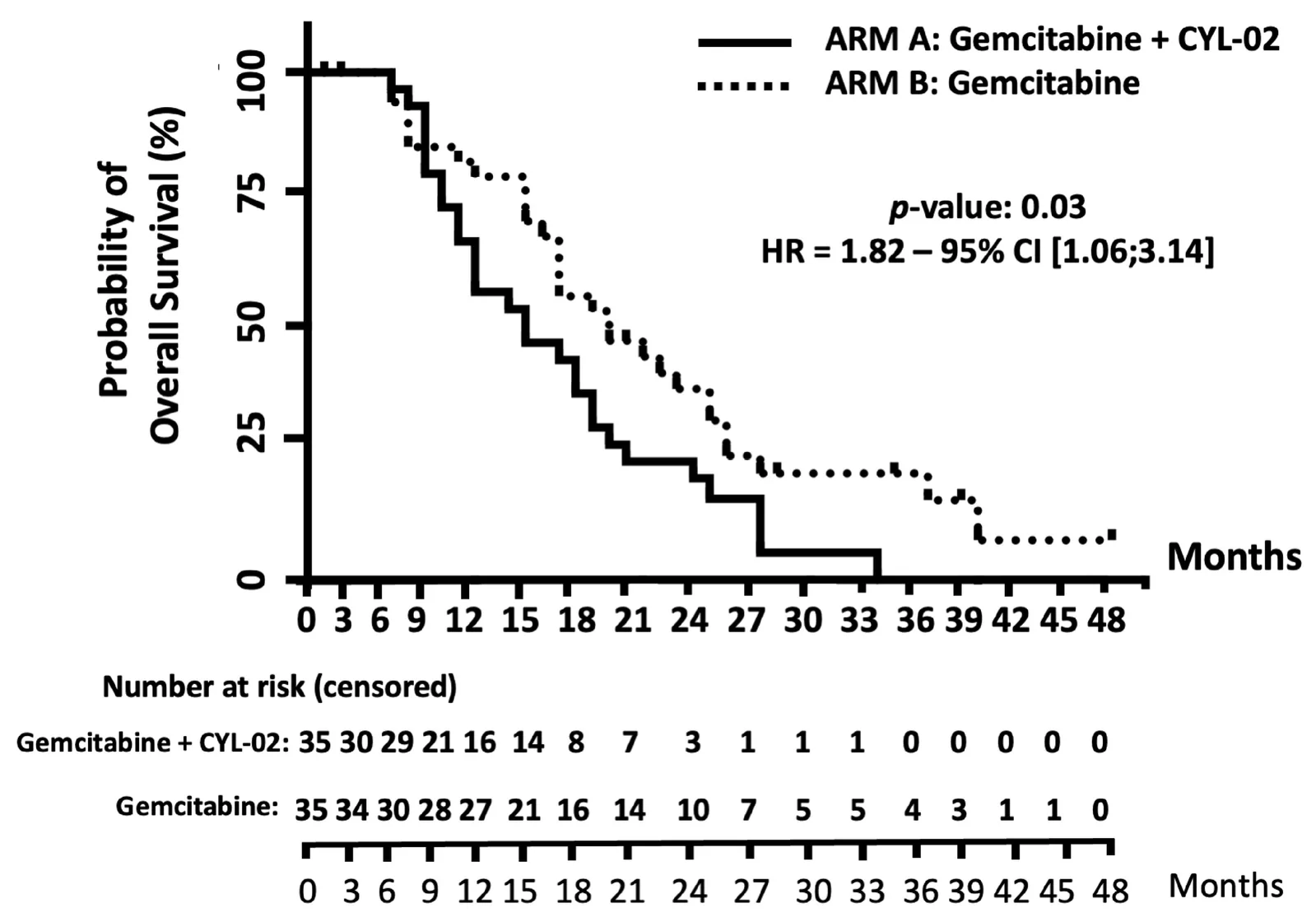
OS analysis using Kaplan-Meier curves for the ITT population of patients with locally advanced PDAC. This figure presents OS probabilities over 48 months for patients treated with CYL-02 + gemcitabine (Arm A, continuous line) and gemcitabine alone (Arm B, dotted line). OS: Overall survival; ITT: intent-to-treat; PDAC: pancreatic ductal adenocarcinoma; HR: hazard ratio; CI: confidence interval.

**Table 2:** Results for primary and secondary endpoints of the Thergap-2 trial

**Data**	**Arm A** **CYL-02 + gemcitabine** ***N* = 35**	**Arm B** **Gemcitabine alone** ***N* = 35**	***P***
**PFS** Median in months (HR; 95%CI) Treated population ITT population Per-protocol population	8.9 9.2 9.4	10.2 (1.16; 0.70-1.92) 10.4 (1.16; 0.70-1.92) 10.8 (1.12; 0.66-1.92)	0.6 0.6 0.75
**OS** Median in months (HR; 95%CI) Treated population ITT population	12.8 13.2	17.5 (1.83; 1.06-3.16) 17.8 (1.82; 1.06-3.14)	0.03 0.03
Tumour response* Progressive disease Partial response Stable disease	2 (6.8%) 1 (3.4%) 26 (89.6%)	3 (9.0%) 4 (12.1%) 26 (78.7%)	0.36
**Tumour size in mm at day 28 *vs.* day 1** Assessed by EUS Mean (SD)	35.2 (10) *vs.* 34.6 (8.2) (median: 33 *vs.* 35)	-	1.0
CA 19-9 level (UI/mL)** Month 2 Month 4 Month 6	301 ± 476 248 ± 437 267 ± 611	286 ± 588 349 ± 537 303 ± 486	0.92 0.62 0.29
Quality of life C-30*** Global health status Physical functioning Role functioning Emotional functioning Cognitive functioning Social functioning Fatigue Nausea, vomiting Pain Dyspnea Insomnia Appetite loss Constipation Diarrhea Financial difficulties	58 (33; 100) 80 (47; 100) 67 (17; 100) 83 (33; 100) 92 (67; 100) 83 (17; 100) 33 (0; 67) 0 (0; 100) 25 (0; 83) 0 (0; 67) 0 (0; 67) 33 (0; 100) 0 (0; 100) 0 (0; 67) 0 (0; 33)	50 (25; 100) 83 (40; 100) 67 (0; 100) 79 (50; 100) 83 (50; 100) 83 (0; 100) 39 (0; 100) 0 (0; 33) 25 (0; 67) 0 (0; 100) 17 (0; 100) 33 (0; 100) 33 (0; 100) 0 (0; 100) 0 (0; 67)	0.85 0.27 0.76 1.00 0.81 0.79 0.13 0.90 0.71 0.65 0.64 0.20 0.27 0.42 0.75

Treated population: all treated patients (Arm A, *n* = 31; Arm B, *n* = 34); ITT population: all randomized patients (Arm A, *n* = 35; Arm B, *n* = 35); Per-protocol population: patients without violations of inclusion and non-inclusion criteria and receiving at least 3 weeks of gemcitabine (Arms A and B) as well as two CYL-02 intra-tumour injections (Arm A) (Arm A, *n* = 26; Arm B, *n* = 31). HR reference Arm B. Survival was evaluated by Log-Rank test.*Missing data: Arm A, *n* = 6; Arm B, *n* = 2. **Missing data: Arm A, *n* = 15; Arm B, *n* = 10; mean ± SD. ***Score at 2 months; complete evaluation assessed in 18 and 22 patients of Arms A and respectively; median score (min.; max.); Values of QOL scores range from 0 to 100. Unpaired and paired Student’s *t* tests, and Fisher’s exact tests were used to determine the statistical significance of differences between two groups of patients. PFS: Progression-free survival; HR: hazard ratio; CI: confidence interval; ITT: intent-to-treat; OS: overall survival; EUS: endoscopic ultrasound; SD: standard deviation; QOL: quality of life.

### Secondary endpoints

EUS-guided CYL-02 intratumoral injections were feasible with no technical failures or delays across all centres, despite involving up to 10 endoscopists. OS was significantly higher in Arm B regardless of the population analysed (treated: 17.5 *vs.* 12.8 months, *P* = 0.03; ITT: 17.8 *vs.* 13.2 months, *P* = 0.03; Table 2). No significant differences were observed between the arms in tumour size (CT scans), tumour progression status, CA 19-9 levels, or quality-of-life parameters [Table 2].

To investigate the unexpected poorer OS in Arm A, we conducted exploratory univariate [Table 3] and multivariate analyses [Table 4]. The ECOG Performance Status at the beginning of the follow-up phase was balanced across both groups [Table 3], indicating that clinical status did not significantly influence the decision to administer second-line treatment based on the assigned group. Indeed, the proportion of patients receiving a complete course of second-line treatment was comparable between the two groups (68.5% *vs.* 77.1%, *P* = 0.59) [Table 3]. The only significant difference observed was the more frequent use of the FOLFOX regimen as a second-line treatment in Arm B [Table 3]. We also performed univariate and multivariate analyses, with the model being adjusted for the factors of randomization such as age and ECOG Performance Status, as well as primary tumour location [Table 4]. The results confirmed that OS remained significantly longer in Arm B compared to Arm A. Similar results were observed when evaluating OS in patients who received second-line treatment [Table 4]. This analysis aimed to minimize immortal time bias and the overestimation of survival by excluding patients who either died during the “protocol phase” or did not receive specific second-line treatment (i.e., those who received only best supportive care). Regardless of the model used, tumor location had no influence on OS [Table 4]. Additionally, in the subgroup of patients who received second-line treatment, the one-year and two-year survival rates were 58.3% and 19.4%, respectively, for patients in Arm A, compared to 85.2% and 35.9% for patients in Arm B.

**Table 3:** ECOG performance status at the beginning of the follow-up phase of the Thergap-2 trial and second-line treatments administered during this phase

**Data**	**Arm A** **CYL-02 + gemcitabine** ***N* = 35**	**Arm B** **Gemcitabine alone** ***N* = 35**	***P***
ECOG Performance status at the beginning of the follow-up phase* 0-1 2	28 (80.0%) 7 (20.0%)	29 (82.8%) 6 (17.2%)	1.00
Second-line treatments** No treatment***	24 (68.7%) 11 (31.4%)	27 (77.1%) 8 (22.9%)	0.59
Types of second-line treatments Radio-chemotherapy**** Folfox Gemcitabine Others*****	8 (33.3%) 4 (16.7%) 6 (25.0%) 6 (25.0%)	10 (37.0%) 13 (48.1%) 2 (7.4%) 2 (7.4%)	0.85 0.02 0.12 0.12

*At the end of the protocol phase. **At least one complete course of second-line chemotherapy or radio-chemotherapy. ***Best supportive care. ****Concomitant oral 5FU and radiotherapy. *****Folfirinox, Capecitabine only, 5FU plus cisplatin. Fisher’s exact tests were used to determine the statistical significance of differences between two patient groups. ECOG: Eastern Cooperative Oncology Group; 5FU: 5-fluorouracile.

**Table 4:** Univariate and multivariable analysis of prognostic factors for OS present at study inclusion

**Factors**	**Number** **event/total (%)**	**HR (95%CI)**	***P***
**Total population (*N* = 70)**			
**Univariate model** Arm B: gemcitabine alone Arm A: CYL-02 + gemcitabine	28/35 (80.0%) 28/35 (80.0%)	(Reference) 1.82 (1.06-3.10)	0.017
**Multivariable model** Arm B: gemcitabine alone Arm A: CYL-02 + gemcitabine	28/35 (80.0%)28/35 (80.0%)	(Reference) 2.09 (1.15-3.80)	0.015
Location of the tumour Head/isthmus Body/tail	39/49 (79.5%) 17/21 (80.9%)	(Reference) 0.69 (0.37-1.31)	0.225
**Patients with a second-line treatment (*N* = 51) (analysed without immortal time bias)**			
**Univariate model** Arm B : gemcitabine alone Arm A: CYL-02 + gemcitabine	22/24 (91.6%) 1/27 (77.7%)	(Reference) 2.27 (1.12-4.62)	0.023
**Multivariate model** Arm B: gemcitabine alone Arm A: CYL-02 + gemcitabine	22/24 (91.6%) 21/27 (77.7%)	(Reference) 2.32 (1.14-4.73)	0.020
Location of the tumour Head/isthmus Body/tail	29/34 (85.2%) 14/17 (82.3%)	(Reference) 0.75 (0.37-1.54)	0.425

*P*-value from Cox model adjusted for Age, ECOG performance, and tumour location. OS: Overall survival; HR: hazard ratio; 95%CI: 95% confidence interval; ECOG: Eastern Cooperative Oncology Group.

### Safety

Supplementary Table 1 reports AEs, regardless of grade, that were considered probably related to treatment. Events are categorized by organ system and treatment arm. In addition, serious AEs with grade ≥ 3 are also detailed in Supplementary Table 2. Safety profiles were comparable, with no treatment-related deaths in either arm. During the protocol phase, 80.7% of patients in Arm A experienced at least one SAE of grade ≥ 3, compared to 64.7% in Arm B (*P* = 0.17). In Arm A, 61.3% of patients experienced at least one grade ≥ 3 SAE related to CYL-02 and/or gemcitabine, with 19.4% (6 patients) experiencing SAEs related to CYL-02 alone: 3 with isolated fever, 1 with increased CRP, and 2 with mild acute pancreatitis. Gemcitabine infusion was delayed in only one patient. In Arm B, 52.9% of patients had at least one grade ≥ 3 SAE related to gemcitabine. During the follow-up phase (median 39.33 months), 32.3% of patients in Arm A and 44.1% in Arm B experienced at least one SAE of any grade.

### CLU is induced in patients receiving CYL-02 and gemcitabine

To identify mechanisms underlying the lack of therapeutic efficacy, we performed CHIP-MS/MS profiling on plasma samples from Thergap-1 patients (NCT01274455)^[18]^. We identified 70 treatment-modulated proteins [Supplementary Tables 3-6], with 8 consistently overexpressed across all doses [Figure 5A and B]. We prioritized CLU based on its known role in chemoresistance in PDAC^[19]^. ELISA validation in Thergap-1 confirmed significant CLU induction (baseline: 90.6 × 10^3^ ± 33.7 × 10^3^ pg/mL *vs.* post-treatment: 299.9 × 10^3^ ± 108.5 × 10^3^ pg/mL, *P* = 0.05, Figure 5C). In Thergap-2, baseline CLU levels were lower in Arm A than Arm B (12.4 × 10^4^ ± 29.9 × 10^3^ *vs.* 18.4 × 10^4^ ± 18 × 10^3^ pg/mL, *P* = 0.04, Supplementary Figure 1A). Gemcitabine alone had no significant impact on CLU (0.7 ± 0.16-fold, Figure 5D and Supplementary Figure 1B), while CYL-02 + gemcitabine induced a 3-fold increase (*P* = 0.0095, Figure 5D and Supplementary Figure 1C).

**Figure 5:**
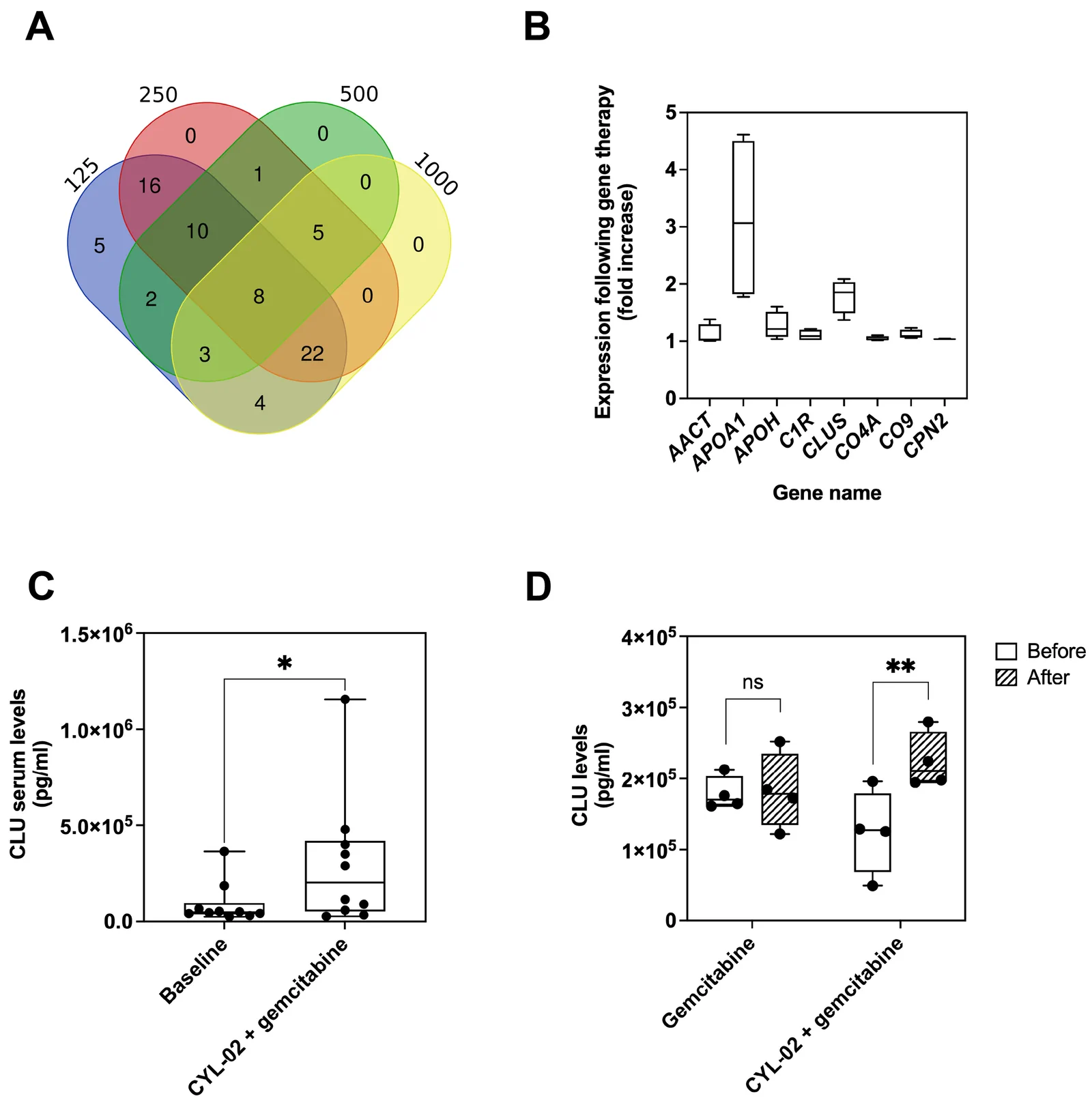
Plasma protein profiling and CLU quantification in Thergap trials. (A) Venn diagram of CHIP-MS/MS profiling of plasma proteins from PDAC patients enrolled in the Thergap-1 trial, at the indicated therapeutic DNA doses; (B) Change in plasma levels of proteins upregulated by CYL-02 + gemcitabine treatment during the Thergap-1 clinical trial. Data are presented as minimum to maximum values (*n* = 24 patients); (C) Analysis of CLU expression by ELISA in the plasma of Thergap-1 patients (*n* = 10), comparing baseline levels and 28 days post-treatment. Data are presented as minimum to maximum values. Statistical analysis was conducted using a paired *t*-test; (D) Analysis of CLU expression by ELISA before and after 2 weeks of treatment in patients enrolled in the Thergap-2 clinical trial (*n* = 4). Data are presented as minimum to maximum values. Statistical analysis was conducted using a Mann-Whitney test. ^*^P < 0.05, ^**^P < 0.01, ns, not significant. CLU: Clusterin; CHIP-MS/MS: microfluidic chip mass spectrometry/tandem mass spectrometry; PDAC: pancreatic ductal adenocarcinoma; ELISA: enzyme-linked immunosorbent assay.

### CLU inhibition enhances gemcitabine response in pancreatic cancer models using the DCK gene from CYL-02

To investigate the causal role of CYL-02 and gemcitabine treatment in CLU upregulation, we developed a cellular model of CYL-02 gene therapy by generating MIA PaCa-2 cells stably overexpressing DCK using lentiviral vectors. These cells were then subjected to chemotherapy. Treatment with gemcitabine alone induced a modest upregulation of CLU expression in MIA PaCa-2 cells (0.21 ± 0.87 *vs.* 0.007 ± 0.06, *P* = 0.02; Figure 6A and Supplementary Figure 2A). This observation was further validated in BxPC-3 [Supplementary Figure 2B] and ASPC-1 cells [Supplementary Figure 2C]. In contrast, DCK overexpression combined with gemcitabine treatment led to a 30-fold increase in CLU expression in MIA PaCa-2 cells (2.72 ± 0.4 *vs.* 0.007 ± 0.06 arbitrary units, *P* = 0.018; Figure 6A and Supplementary Figure 2A).

**Figure 6:**
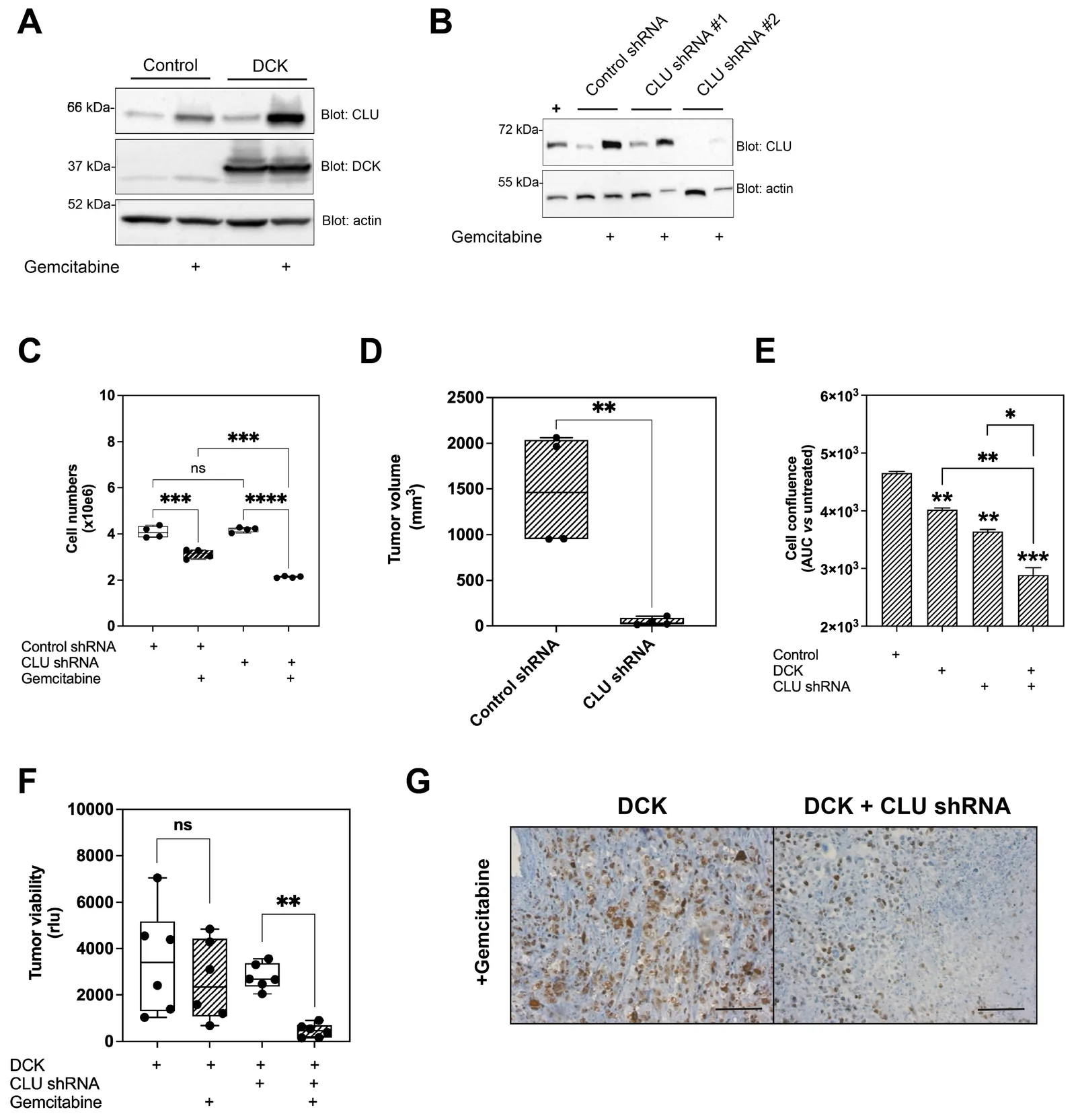
CLU inhibition enhances gene therapy and chemotherapy efficacy in experimental models of PDAC. (A) Western blot for CLU and DCK expression in Mia PaCa-2 cells stably expressing DCK. Mia PaCa-2 cells stably expressing GFP were used as a control. Cells were treated with or without 100 nM gemcitabine for 48 h. PBS was used as a mock treatment. Actin was used as a loading control. Data are representative of three biological replicates (*n* = 3); (B) Western blot for CLU expression in Mia PaCa-2 cells expressing control shRNAs or shRNAs targeting CLU. Actin was used as a loading control. Data are representative of three biological replicates (*n* = 3); (C) Cell counting of Mia PaCa-2 cells expressing either control shRNA or CLU shRNA, treated with or without 100 nM gemcitabine. PBS was used as a mock treatment. Results represent the mean of four independent experiments, each performed in triplicate. Statistical analysis was conducted using a two-way ANOVA; (D) Tumor volume of Mia PaCa-2 orthotopic xenografts in NSG mice expressing either control shRNA or CLU shRNA, treated or not with gemcitabine (*n* = 4 per group). Data are presented as minimum to maximum values. Statistical analysis was performed using a two-way ANOVA; (E) Cell confluence of Mia PaCa-2 cells expressing control shRNA, CLU shRNA, and/or DCK, treated with or without 100 nM gemcitabine. PBS was used as a mock treatment. Results represent the mean of four independent experiments, each performed in triplicate. Statistical analysis was conducted using a two-way ANOVA; (F) Tumour viability of Mia PaCa-2 orthotopic xenografts in NSG mice expressing control shRNA, CLU shRNA, and/or DCK, treated or not with gemcitabine (*n* = 6 per group). PBS was used as mock treatment. Data are presented as minimum to maximum values. Statistical analysis was performed using a two-way ANOVA; (G) Ki67 staining in Mia PaCa-2 orthotopic xenografts expressing DCK and/or CLU shRNA, treated with gemcitabine. Images are representative of 15 fields from 3 different tumours. Scale bar: 100 µm. ^*^P < 0.05, ^**^P < 0.01, ^***^P < 0.005, ^****^P < 0.001, ns, not significant. CLU: Clusterin; PDAC: pancreatic ductal adenocarcinoma; GFP: green fluorescent protein; PBS: phosphate-buffered saline; shRNAs: small hairpin RNAs; ANOVA: analysis of variance; AUC: area under the curve.

Considering the well-established role of CLU in chemotherapy resistance, particularly in PDAC^[19]^, where it inhibits gemcitabine-induced apoptosis and thereby reduces drug efficacy, we investigated whether targeting CLU could further sensitize cancer cells to combined DCK gene therapy and chemotherapy. We then generated CLU-silenced MIA PaCa-2 cells using small hairpin RNA (shRNA). Western blot analysis indicated that two independent shRNAs (CLU shRNA #1 and #2) efficiently inhibited both basal and gemcitabine-induced CLU expression in cells [Figure 6B] and CLU secretion into the culture medium [Supplementary Figure 2D]. Subsequent experiments used CLU shRNA #2, hereafter referred to as CLU shRNA, which exhibited the highest silencing efficacy.

CLU knockdown had no effect on proliferation alone but significantly sensitized cells to gemcitabine (-48% ± 1% *vs.* -23% ± 3% in controls, *P* < 0.005, Figure 6C). To further assess the impact of CLU inhibition *in vivo*, MIA PaCa-2 cells expressing CLU shRNA or control shRNA were orthotopically implanted into the pancreas of NSG mice. Mice received 135 mg/kg of gemcitabine every 2-3 days for 9 days, and tumours were harvested, measured, and weighed at the experimental endpoint. CLU silencing enhanced gemcitabine’s antitumor effects, reducing tumour volume by 69% ± 25% (*P* < 0.01, Figure 6D) and weight by 72% ± 11% (*P* < 0.005, Supplementary Figure 2E). To further assess whether CLU inhibition enhances DCK-mediated chemosensitization, we generated MIA PaCa-2 cells stably overexpressing DCK and expressing CLU shRNA. Combining DCK overexpression with CLU silencing produced superior chemosensitization and a 38% reduction in cell confluence *vs.* 14% with DCK alone (*P* < 0.01; Figure 6E). We then orthotopically engrafted MIA PaCa-2 cells overexpressing DCK and/or expressing CLU shRNA into the pancreas of NSG mice. Control cells expressed DCK and a non-targeting shRNA. All cell lines expressed luciferase to allow tumour viability monitoring. Gemcitabine treatment significantly reduced tumour volume in DCK-overexpressing tumours (-26% ± 25%, *P* < 0.01) and tumours co-expressing DCK and CLU shRNA (-24% ± 25%, *P* < 0.005) compared to control tumours [Supplementary Figure 2F]. However, CLU silencing did not further enhance the effect of DCK on tumour volume reduction [Supplementary Figure 2F]. In contrast, co-expression of DCK with CLU shRNA led to a marked reduction in tumor viability, as monitored by cancer cell-derived bioluminescence, when combined with gemcitabine (-83% ± 25%, *P* < 0.01, Figure 6F). Consistent with these findings, Ki67 staining, used as a marker of cancer cell proliferation, revealed that CLU targeting significantly decreased tumour cell proliferation compared to DCK overexpression alone in tumours treated with gemcitabine (11% ± 4% *vs.* 40% ± 8%, *P* < 0.001, Figure 6G and Supplementary Figure 2G). Together, these findings demonstrate that CLU inhibition enhances the chemosensitizing effect of DCK, providing a rationale for combining CYL-02 gene therapy with CLU-targeting strategies to overcome resistance to treatment in PDAC.

## DISCUSSION

This study represents one of the few gene therapy trials to combine intratumoral therapeutic gene delivery with a multicentre Phase 2 trial design for patients with locally-advanced PDAC. We demonstrated the feasibility of this approach, with no AEs attributable to the endoscopic procedure, despite the logistical complexity of a multicentre setting. Previous studies have also explored intratumoral gene delivery in multicentre phase II trials across various tumour types^[20-22]^. In addition, intratumor injection of gene therapy product in PDAC via endoscopic ultrasound (including our previous experience) has also demonstrated good feasibility and tolerability in a Phase 1-2 design^[18,23-25]^. Collectively, these studies support the clinical viability of ultrasound-guided intratumoral gene therapy in multicentre settings.

Unfortunately, the Thergap-2 trial did not meet its primary endpoint of improved PFS with the addition of CYL-02 to gemcitabine in patients with locally advanced PDAC. The worse OS in the CYL-02 + gemcitabine arm is concerning and warrants careful interpretation. We found no differences in treatment exposure, baseline characteristics, or subgroup effects that could account for this finding. Patients’ clinical status was comparable between the two arms, and no differences in AEs were observed during follow-up. As such, the number of second-line treatments was administered equally in both groups; however, the types and durations of these second-line therapies varied across centres and may have impacted OS. Indeed, a recent Phase 3 study in patients with locally advanced PDAC reported that the PFS benefit of FOLFIRINOX over gemcitabine did not translate into an OS advantage, likely due to the influence of second-line regimens^[26]^. In our study, subsequent management was left to the prescriber’s discretion and was not constrained by the protocol. While the gemcitabine group received FOLFOX as second-line therapy more frequently (48.1% *vs.* 16.6%, *P* = 0.02), the overall proportion of patients receiving any second-line treatment, as well as the baseline ECOG Performance Status, were comparable between the groups. In addition, using a multivariate model adjusted for age, ECOG Performance Status, and tumor location, OS remained significantly longer in the gemcitabine group compared to the CYL-02 + gemcitabine group. Consequently, no clear clinical factors were identified that could explain the observed discrepancy in OS.

This negative clinical outcome paradoxically served as a critical starting point for uncovering a biologically meaningful resistance mechanism. Through systematic ancillary proteomic analyses, we identified CLU as a treatment-induced factor consistently upregulated in patients receiving CYL-02 + gemcitabine. Moreover, we demonstrate a direct link between CLU expression and CYL-02, as DCK expression is sufficient to drive CLU upregulation in response to gemcitabine *in vitro*. CLU has been extensively documented to be induced in cancer cells undergoing treatment and associated with resistance to ferroptosis^[27]^ and MEK inhibitors^[28]^, and, historically, to gemcitabine^[19]^, particularly in PDAC-derived cells. We extended this observation to BxPC-3 cells and AsPC-1 cells, confirming that CLU induction by gemcitabine is conserved across PDAC molecular subtypes. We therefore hypothesized that its induction by the combination of CYL-02 and gemcitabine may have promoted tumor cell survival, thereby counteracting the intended chemosensitization effect.

Furthermore, we demonstrated that targeting CLU restores chemotherapy efficacy in experimental PDAC models, consistent with previously published work^[29]^. While we cannot exclude the involvement of other resistance pathways, especially involving CDA expression and function^[17]^, the magnitude and consistency of the CLU signal observed in patients, combined with the reversibility data from *in vitro* and *in vivo* models, suggest a potential central role for CLU in mediating resistance to this gene therapy approach. These findings may have immediate clinical relevance. Targeting CLU with custirsen (OGX-011), a second-generation antisense oligonucleotide^[30]^, represents a promising strategy to overcome adaptive resistance to CYL-02 and gemcitabine treatment. This approach aligns with the growing recognition that adaptive resistance mechanisms may be as important as intrinsic resistance in limiting the therapeutic efficacy in PDAC.

We acknowledge several limitations in this study. First, ELISA quantification of plasma CLU was performed on a small subset of trial participants, which significantly limits the statistical power to draw robust clinical conclusions and must be interpreted as preliminary exploratory signals. As a result, we were unable to statistically correlate CLU expression levels with patient survival. Definitive clinical validation will require prospective collection of larger, independent patient cohorts in future trials. However, the consistent upregulation of CLU observed across both Thergap-1 and Thergap-2 subsets reinforces the reliability of this preliminary observation and warrants further investigation. In addition, our preclinical models used a single cell line and immunodeficient mice, which do not fully recapitulate PDAC heterogeneity or the tumour microenvironment. Nevertheless, using the same therapeutic genes (DCK) and drug (gemcitabine) as in the clinical trial enhances translational relevance. Moreover, PDAC is an intrinsically complex tumor whose biology is profoundly influenced by a heterogeneous and dynamic microenvironment^[8]^. This complexity not only contributes to its aggressive clinical phenotype but also drives its persistent resistance to both conventional and emerging therapeutic strategies^[8]^. Interestingly, a recent study identified a subset of cancer-associated fibroblasts (CAFs) expressing CLU in PDAC, which are associated with immune-regulatory functions in BRCA-mutated tumours following activation of heat-shock factor 1^[31]^. This raises the question of whether CLU expression and its functional role in the PDAC stroma are modulated by CYL-02 and gemcitabine treatment. Further investigations are warranted, particularly given that CLU expression has been shown to prevent macrophage polarization toward the pro-inflammatory M1 phenotype, a process linked to the reactivation of anti-tumour immunity in breast cancer^[32]^.

In conclusion, the Thergap-2 trial highlights the critical role of adaptive resistance in limiting the efficacy of gene therapy for PDAC. By identifying CLU as a treatment-induced resistance factor and demonstrating that its inhibition restores chemosensitivity, our study transforms the findings of a negative trial into a biologically insightful roadmap for future combination strategies. These findings provide a strong rationale for targeting adaptive resistance mechanisms to improve innovative therapy outcomes in this devastating disease.

## DECLARATIONS

### Availability of data and materials

The data that support the findings of this study as well as the full statistical analysis are available from the corresponding authors upon reasonable request.

### AI and AI-assisted tools statement

During the preparation of this manuscript, the AI tool L’Assistant (version 0.0.19) was used solely for language editing. The tool did not influence the study design, data collection, analysis, interpretation, or the scientific content of the work. All authors take full responsibility for the accuracy, integrity, and final content of the manuscript.

### Copyright

© The Author(s) 2026.
