# Supplementary material for: Randomized multicentre Phase 2 clinical trial of non-viral gene therapy for pancreatic cancer: clinical and molecular findings
Source: Cancer Drug Resist. 2026 Oct 22;9:38. doi: 10.20517/cdr.2026.68 (PMC13749215; doi:10.20517/cdr.2026.68)
Supplement: Supplementary file 1 [file cdr-9-38-SupplementaryMaterials.zip › cdr9068-SupplementaryMaterials/cdr9068-Supplementary Figures.pdf]

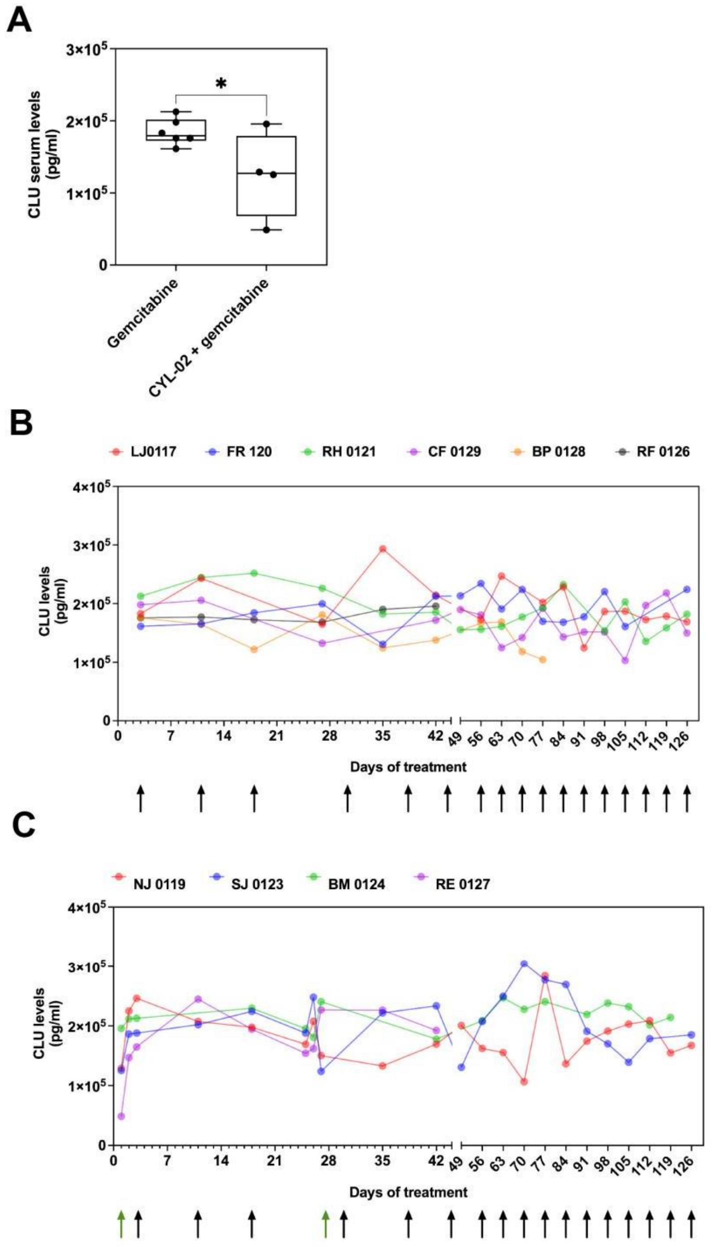

Supplementary Figure 1. Clusterin dectection in Thergap-2 patients. (A) Clusterin (CLU) was detected by ELISA at baseline in Thergap-2 patients from arm A (gemcitabine, n=6) and arm B (CYL-02 + gemcitabine, n=4). Data are presented as minimum to maximum values. Statistical analysis was conducted using an unpaired t-test. Plasmatic CLU detection by ELISA during the course of the treatment in PDAC patients from Thergap-2 clinical trial receiving gemcitabine only (A, n=6) or CYL-02 + gemcitabine (B, n=4). Arrows indicate gemcitabine (black) and intratumoral CYL-02 (green) administration. Patient IDs are indicated. *p<0.05.

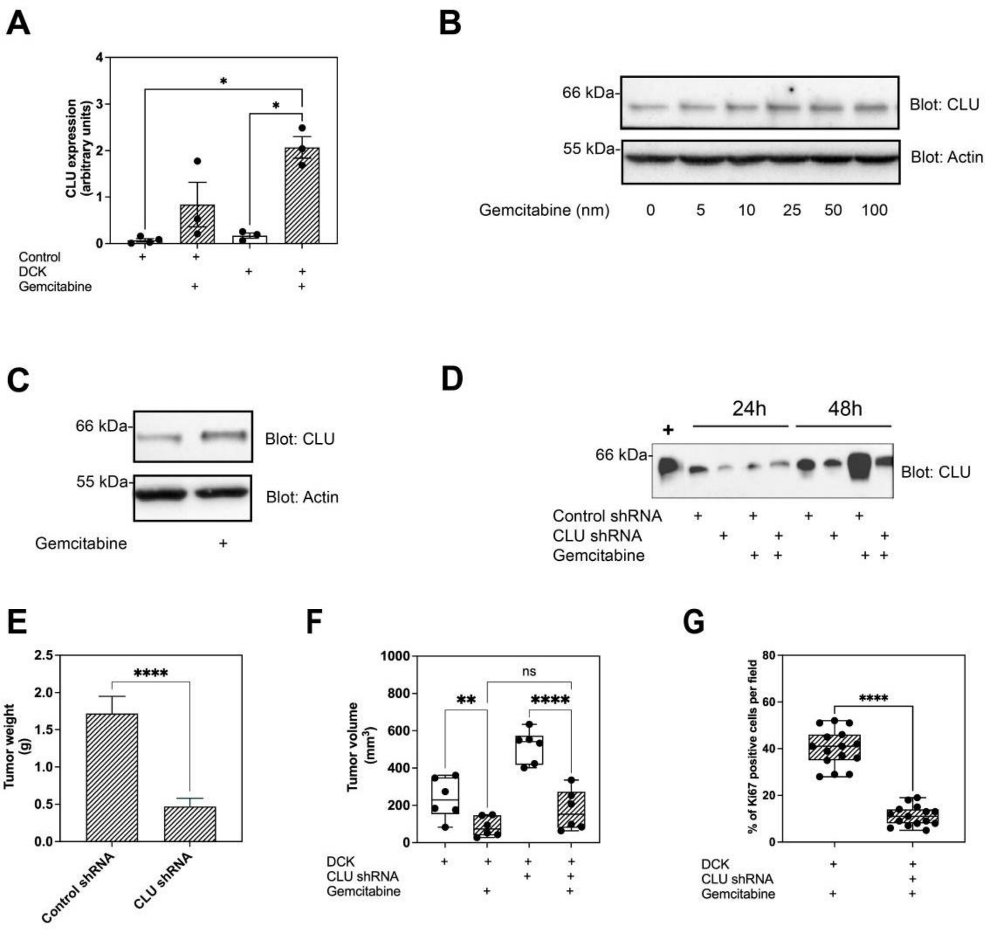

Supplementary Figure 2. Clusterin inhibition enhances gene therapy and chemotherapy efficacy in experimental models of PDAC. (A) Quantification of CLU and DCK expression by Western blot in Mia PaCa-2 cells expressing DCK or GFP as a control, and treated or not with 100 nM gemcitabine for 48 hours. PBS was used as a mock treatment. Actin was used as a loading control. Data represent the mean ± SD of three biological replicates (n=3). Statistical analysis was conducted using a two-way ANOVA. (B) Quantification of CLU expression by Western blot in BXPC-3 cells treated or not with gemcitabine for 48 hours at the indicated dose. PBS was used as a mock treatment. Actin was used as a loading control. Data represent two biological replicates (n=2). (C) Quantification of CLU expression by Western blot in ASPC-1 cells treated or not with 50 nM gemcitabine for 48 hours. PBS was used as a mock treatment. Actin was used as a loading control. Data represent two independent experiments. (D) Western blot analysis of CLU in the cell supernatant of Mia PaCa-2 cells expressing control or CLU shRNA, treated or not at the time indicated with 100 nM gemcitabine. PBS was used as a mock treatment. Data are representative of four biological replicates (n=4). (E) Tumor weight at the endpoint in Mia PaCa-2 xenografts in NSG mice expressing control or CLU shRNA, treated with gemcitabine. Data are presented as mean + SD (n = 4 mice per group). Statistical analysis was performed using a two-way ANOVA. (F) Tumor volume at the endpoint in Mia PaCa-2 xenografts expressing DCK and control or CLU shRNA, treated or not with gemcitabine PBS was used as a mock treatment. Data are presented as min-max (n =5

mice per group). Statistical analysis was conducted using a two-way ANOVA. (G) Quantification of Ki67 staining at the endpoint in Mia PaCa-2 xenografts in NSG mice expressing DCK and control or CLU shRNA, treated with gemcitabine. Data are representative of 15 fields from 3 independent tumors (min-max). Statistical analysis was performed using an unpaired t-test. * $p<0.05$, ** $p<0.01$, **** $p<0.001$.
