# Supplementary material for: Randomized multicentre Phase 2 clinical trial of non-viral gene therapy for pancreatic cancer: clinical and molecular findings
Source: Cancer Drug Resist. 2026 Oct 22;9:38. doi: 10.20517/cdr.2026.68 (PMC13749215; doi:10.20517/cdr.2026.68)
Supplement: Supplementary file 1 [file cdr-9-38-SupplementaryMaterials.zip › cdr9068-SupplementaryMaterials/cdr9068-Supplementary Table 1.pdf]

SUPPLEMENTAL TABLE 1: Adverse events whatever their grade probably related to the treatments and that occurred during the treatment phase of the Thergap-2 trial

**SERIOUS AVERSE EVENTS WHATEVER THEIR GRADE BY SYSTEM ORGAN CLASS**	**ARM A** CYL02 + Gemcitabine N = 31	**ARM B** Gemcitabine alone N = 34	*p*
Cardiologic	0 (0 %)	1 (2.94%)	*1.0*
Skin	7 (22.58 %)	6 (17.65%)	*0.75*
Kidney and urinary tract	2 (6.45 %)	0 (0 %)	*0.22*
Immune system	1 (3.23 %)	0 (0 %)	*1.0*
Nervous system	5 (16.13 %)	6 (17.65%)	*1.0*
Gastrointestinal	23 (74.19 %)	28 (82.35%)	*0.54*
Hematologic	26 (83.87%)	28 (82.35%)	*1.0*
Hepatobiliary	6 (19.35 %)	6 (17.65%)	*1.0*
Musculoskeletal	3 (9.68%)	2 (5.88%)	*0.66*
Ocular	1 (3.23 %)	2 (5.88%)	*1.0*
Vascular	3 (9.68%)	1 (2.94%)	*0.34*
Respiratory	2 (6.45 %)	2 (5.88%)	*1.0*
Psychiatric	0 (0 %)	1 (2.94%)	*1.0*
Metabolism and nutrition	13 (41.94 %)	13 (38.24%)	*0.8*
General status (*)	25 (80.65 %)	28 (82.35%)	*1.0*
Biology (**)	23 (74.19 %)	26 (76.47%)	*1.0*

* : Fever, fatigue, flue syndrome, edema, pain

** : ALAT, ASAT, Gamma Glutamyl transferase, blood cells counts, platelets, lipase, glucose, LDH, sodium, potassium, urea, creatinine, iron, albumin, protein, CRP
