# Supplementary material for: Randomized multicentre Phase 2 clinical trial of non-viral gene therapy for pancreatic cancer: clinical and molecular findings
Source: Cancer Drug Resist. 2026 Oct 22;9:38. doi: 10.20517/cdr.2026.68 (PMC13749215; doi:10.20517/cdr.2026.68)
Supplement: Supplementary file 1 [file cdr-9-38-SupplementaryMaterials.zip › cdr9068-SupplementaryMaterials/cdr9068-Supplementary Table 2 .docx]

**SUPPLEMENTAL TABLE 2 : Serious adverse events with a grade ≥ 3 probably related to the treatments and that occurred during the treatment phase of the Thergap-2 trial.** * : Fever, fatigue, flue syndrome, edema, ** Investigations: ALAT, ASAT, Gamma Glutamyl transferase, blood cells count, platelets, lipase, CRP

| **SERIOUS AVERSE EVENTS WITH GRADE ≥ 3 BY**  **SYSTEM ORGAN CLASS** | **ARM A**  CYL02 + Gemcitabine  N = 31 | **ARM B**  Gemcitabine alone  N = 34 | *p* |
| --- | --- | --- | --- |
| Skin | 0 (0 %) | 1 (2.94%) | *1.0* |
| Gastrointestinal | 4 (12.90 %) | 3 (8.82 %) | *0.7* |
| Hematologic | 6 (19.35 %) | 9 (26.47%) | *0.56* |
| Hepatobiliary | 1 (3.23 %) | 2 (5.88%) | *1.0* |
| Vascular | 0 (0 %) | 1 (2.94%) | *1.0* |
| Metabolism and nutrition | 1 (3.23 %) | 3 (8.82 %) | *0.61* |
| General status (*) | 6 (19.35 %) | 5 (14.71%) | *0.74* |
| Biology (**) | 23 (74.19 %) | 26 (76.47%) | *1.0* |
